# A Modified Shielding and Rapid Transition DDES Model for Separated Flows

**DOI:** 10.3390/e25040613

**Published:** 2023-04-04

**Authors:** Da Lei, Hui Yang, Yun Zheng, Qingzhe Gao, Xiubo Jin

**Affiliations:** 1 School of Energy and Power Engineering, Beihang University, Beijing 100191, China; 2 Jiangxi Research Institute, Beihang University, Nanchang 330096, China

**Keywords:** delayed detached eddy simulation, boundary layer shielding, modeled stress depletion, RANS-LES transition, gray area mitigation, entropy production

## Abstract

In this paper, the major problems associated with detached eddy simulation (DES) (namely, modeled stress depletion (MSD) and slowing of the RANS to LES transition (RLT)) are discussed and reviewed, and relevant improvements are developed. A modified version for the delayed DES (DDES) method with adaptive modified adequate shielding and rapid transition is proposed; this is called MSRT DDES. The modified shielding strategy can be adjusted adaptively according to the local flow conditions: keeping the RANS behavior in the whole boundary layer when there is no resolved turbulence, and weakening the shielding function when resolved turbulence exists in the mainstream over the boundary layer. This strategy can significantly ameliorate the MSD in the RANS boundary layer, regardless of the mesh refinement, and avoid excessive shielding in the fully developed resolved turbulence that may otherwise delay the development of the separated and reattached flow. Three cases are designed to test the modified DDES, namely, complete shielding in the RANS zone of a boundary layer (the zero-pressure gradient turbulent boundary layer with the refined mesh), modified adaptive improved shielding with a rapid transition (the flow over a hump), and the overall performance in a complex 3D separation (the corner separation in a compressor cascade). The results show that the modified shielding function is more physical than earlier proposals compared to shielding functions, and according to detailed comparisons of the wall skin friction coefficients, velocity profiles, total pressure-loss coefficients, entropy production analyses, and so on, the MSD and RLT problems are moderately alleviated by the MSRT DDES.

## 1. Introduction

One of the most promising hybrid RANS/LEES methods is detached eddy simulation (DES), as proposed by Spalart [1,2]. DES behaves like Reynolds-averaged Navier–Stokes (RANS) simulations in the wall boundary layer, while it exhibits large eddy simulation (LES) behavior far away from the wall. According to the relative sizes of RANS scales and LES scales, the flow field is spatially partitioned into RANS regions and LES regions. DES is considered a weak coupling method [3] and leads to various problems.

Near the interface of RANS and LES regions, the RANS eddy viscosity decreases to the level of the SGS viscosity, and the highly averaged flow field similar to the “laminar flow” can only switch to the resolved turbulence with three-dimensional fluctuations via nonmodal growth mechanisms. This area, where the fluid does not completely transition from RANS behavior to LES behavior, is known as the “gray area” and is formed due to a lack of direct transfer from the modeled turbulence energy to the resolved turbulence energy. The gray area presents two serious problems, which are described below.

### 1.1. Modeled Stress Depletion

When the grid in the wall boundary layer is over-refined, the gray area will be located within the boundary layer. As a result of the delayed generation of resolved turbulence, the resolved Reynolds stress cannot compensate for the reduction in the modeled Reynolds stress; this is the so-called modeled stress depletion (MSD) [4], further leading to grid-induced separation (GIS). The delayed DES (DDES) proposed by Spalart [4] maintains the RANS behavior in the boundary layer and delays the LES behavior via the shielding function $f_{d}$, thus alleviating the MSD problem to a large extent. However, the shielding function $f_{d}$ cannot completely cover the whole boundary layer [5] and will be further weakened in boundary layers with an over-refined mesh or a strong adverse pressure gradient [3,6,7]. The application of artificial turbulence at the inlet can alleviate the MSD problem, in which case the solution will exhibit WMLES behavior [8]. The inner layer of the boundary layer keeps the RANS behavior, and the turbulence level in the outer layer is maintained by the artificial resolved turbulence. However, this will introduce nonphysical phenomena, which require a transition distance to recover a well-behaved turbulent flow. The much higher grid resolution is needed for the WMLES behavior to forecast anisotropy turbulence in near-wall dynamics [9].

Many studies on MSD focused on the remedy of the shielding function. Increasing the protective capability by changing the constants of the shielding function [5,7] is too case-dependent and could cause the excessive shielding of separated flows or free shear flows. Probst et al. [6] constructed a shielding function with an algebraic sensor. In the study of airfoil separated flows, lists of points lying on an approximate wall-normal ray were constructed, so that velocity profiles were obtained to calculate the thickness of the boundary layer. However, it was difficult to find a unified boundary criterion for inner flows and outer flows. In addition, the wall-normal point lists were nonlocal structures that could be unreasonable in complex boundary situations (e.g., corner flows and tip leakage flows). Deck [3] proposed to superimpose a second shielding function on the standard shielding function $f_{d}$ to protect the whole attached boundary layer profile, regardless of the infinitely fine meshes and arbitrary pressure gradients. An inhibition function was introduced to prevent the increased shielding from delaying the formation of instabilities in separated flows or free shear layers. This method achieves complete shielding of the boundary layer without resolved turbulence in the mainstream.

### 1.2. RANS-to-LES Transition (RLT) Problem

Another problem is the RANS-to-LES transition (RLT) problem. Owing to the excessive shielding function and convection of the high eddy viscosity from the upstream RANS region, the development of inviscid instability in the gray area will be inhibited. Even under low eddy viscosity, the RANS “laminar flows” still need space to transit into three-dimensional turbulent flows. These two issues will lead to a serious delay in the development of resolved turbulence downstream of the separated flow, and then lead to discrepancies in physical situations, such as a smaller vorticity thickness [10] in the free shear flow and a delayed reattachment position [11] in the back-step and hump cases. Applying artificial turbulence at the RANS/LES interface in the separation zone might accelerate the 3D turbulence recovery [12,13]. However, it is difficult to ensure the “quality” of artificial turbulent content, especially for complex separated flows.

Most solutions for the RLT problem involve reducing the eddy viscosity in the gray area to reduce dissipation and accelerate the development of the non-viscous instability. Mockett [14] introduced the WALE SGS model [15] and the σ SGS model [16] into DDES. The difference operators of the WALE and σ SGS models can decrease the sub-grid viscosity in the quasi-2D flow regions and recover the normal SGS model activity in the developed 3D turbulence. However, the LES scales are improved to make the sub-grid viscosity level match the resolution of local grids. Chauvet [17] changed the LES scale into the projected length of grids on the plane perpendicular to the local vortex vector. This LES scale, $\Delta_{\omega }$, takes into account the different resolution abilities of a highly anisotropic grid in different directions. On the basis of $\Delta_{\omega }$, the grid length scale $\Delta_{SLA}$ proposed by Shur [18] introduces the vortex tilting measure (VTM) specifically for rapid transition in gray regions. The VTM can distinguish laminar flow regions from 3D turbulence regions, and further reduce the grid length scale in laminar flow regions to accelerate the RLT. Various studies have verified its applicability to different flow phenomena [19,20] other than free shear flows. Moreover, the VTM has the ability to detect the 3D vortex structure and identify the specific areas of flow fields.

### 1.3. Coupling of the MSD and RLT Problems

**Figure 1:**
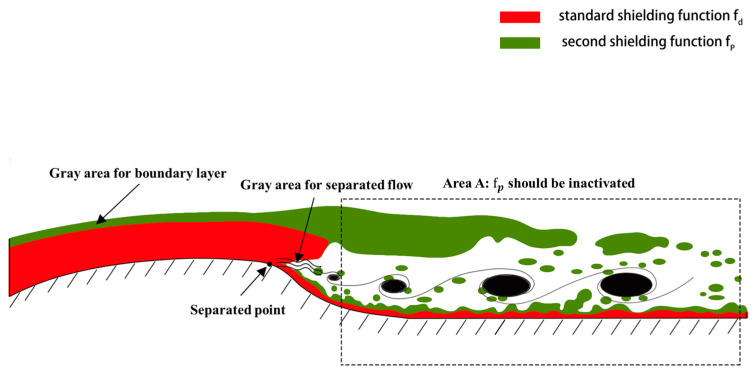
A typical separated–reattached flow.

The MSD and RLT problems are coupled in separated–reattached flows, which makes the previous DES-type model perform poorly near the reattached area. On the premise that artificial turbulence is not applied, DDES is required to maintain the RANS behavior in the entire boundary layer, and quickly switch to LES behavior in flow-separation regions. This idea is more reasonable for a boundary layer outside which there is no resolved turbulence (such as the free shear flow over a flat plate). However, for a reattached boundary layer in the separated–reattached flows, such as the flow over a hump and the back-step flow, maintaining the RANS behavior in the entire boundary layer will be excessive. In the reattached region (Area A in Figure 1), the strong 3D resolved turbulence induced by the flow separation is close to the wall and occupies most areas of the outer layer of the boundary layer. The strong 3D resolved turbulence can alleviate the “gray area” problem of the local boundary layer (the resolved Reynolds stress of LES can compensate for the reduced modeled Reynolds stress of RANS). Therefore, the shielding function should be weakened adaptively in this area. The improved DDES (IDDES) proposed by Shur [21] can switch between DDES and WMLES according to whether there is resolved turbulence in the boundary layers. However, the shielding function in IDDES still cannot guarantee the complete shielding of the boundary layer in the DDES branch, and no means have been developed to alleviate the gray area problem to accelerate the RLT. Francesco De Vanna [22] suggested a novel numerical methodology merging wall-modeled and wall-resolved LES techniques, which introduces an enlightening switching strategy. The dominance of WMLES and WRLES is decided by the inner-scaled wall spacings which are the ratio of the grid spacings to local viscous lengths in the corresponding direction. WMLES will be activated when any one of inner-scaled wall spacings does not reach the redetermined threshold. This strategy considers both the grid resolutions and the local turbulence scales. However, the MSD can still be a problem since the complete shielding in the equilibrium boundary layers cannot be guaranteed.

In this study, a variant of DDES with modified shielding and rapid transition is proposed; this is referred to as MSRT DDES. This variant is designed to ensure that the whole boundary layer is shielded by means of the second shielding function when there is no resolved turbulence in the mainstream; the transition of RANS-LES in the gray area is accelerated by $\Delta_{SLA}$ [18] when flow separation occurs, and the shielding of the boundary layer is weakened adaptively when there is 3D-resolved turbulence in the mainstream, which is realized by the modified shielding function $f_{VTM}$ constructed in this paper. Several test cases are used to examine the MSRT DDES. The shielding capability of $f_{VTM}$ in the boundary layer without resolved turbulence is tested using the case of a zero-pressure gradient turbulent boundary layer over a flat plate with a challenge area. The ability of modified shielding in the MSRT DDES is tested using a wall-mounted hump case with separated–reattached flows. Lastly, the overall performance of the MSRT DDES in respect of a complex three-dimensional flow is tested via a linear compressor cascade with corner separation.

## 2. Proposed Approaches

The governing equations of the SST DDES model are as follows [23]:

(1) $$\frac{\partial \rho k}{\partial t}+\nabla \cdot \left(\rho \vec{U}k\right)=\nabla \cdot \left[\left(\mu +\sigma_{k}\mu_{t}\right)\nabla k\right]+P_{k}-\frac{\rho \sqrt{k^{3}}}{l_{DDES}},$$

(2) $$\frac{\partial \rho \omega }{\partial t}+\nabla \cdot \left(\rho \vec{U}\omega \right)=\nabla \cdot \left[\left(\mu +\sigma_{\omega }\mu_{t}\right)\nabla \omega \right]+2(1-F_{1})\rho \sigma_{\omega 2}\frac{\nabla k\cdot \nabla \omega }{\omega }+\alpha \frac{\rho }{\mu_{t}}P_{k}-\beta \rho \omega^{2}.$$

The DDES length scale in Equation (1) is as follows:

(3) $$l_{DDES}=l_{RANS}-f_{d}\max\left(0,l_{RANS}-l_{LES}\right),$$

(4) $$l_{RANS}=\frac{\sqrt{k}}{C_{\mu }\omega },$$

(5) $$l_{LES}=C_{DES}\Delta ,$$

(6) $$C_{DES}=C_{DES1}\cdot F_{1}+C_{DES2}\cdot (1-F_{1}),$$

where Δ is the grid length scale, and $f_{d}$ is the shielding function. The model constants are identical to those of the original model [23]. The grid length scale and shielding function are modified as described below.

### 2.1. Alternative Grid Length Scales

Taking the hump flow in the XY-plane as an example, the initial region of the separated flow from the wall is generally a gray area, where the flow is quasi-2D. The mesh in this area is generally anisotropic with a scale in the Z-direction larger than that in the X- and Y-directions. In the downstream of separated flows, there is a core area with developed 3D turbulence, and the mesh in that area should be as isotropic as possible for the LES resolution. To enable selection of the most appropriate grid scale, Table 1 shows the length orders of several different grid length scales in the quasi-2D flow regions and the developed 3D turbulence region. The resolution of grids to a quasi-2D flow in an XY-plane should be determined by the grid scales in the X- and Y-directions; thus, ${\tilde{\Delta }}_{\omega }$ and $\Delta_{SLA}$ are more reasonable than the standard $\Delta_{\max}$ and $\Delta_{\mathrm{cube}-\mathrm{root}}$. $\Delta_{SLA}$ reduces the grid length scale for quasi-2D flow regions to accelerate the RANS-LES transition; hence, it is finally applied in the MSRT DDES.

#### 2.1.1. Max Length Scale $\Delta_{\max}$

The typical length scale $\Delta_{\max}$ used in the standard DDES formulation is given by

(7) $$\Delta_{\max}=\max\left(\Delta x,\Delta y,\Delta z\right).$$

#### 2.1.2. Shear Layer Adapted Length Scale $\Delta_{\mathrm{SLA}}$

$\Delta_{SLA}$ [18] is defined as per Equation (8).

(8) $$\Delta_{SLA}={\tilde{\Delta }}_{\omega }F_{KH}\left(<VTM>\right),$$

(9) $${\tilde{\Delta }}_{\omega }=\frac{1}{\sqrt{3}}\underset{n,m=1,8}{\max}\left|\left(I_{n}-I_{m}\right)\right|,\;$$

(10) $$I_{n}=n_{\omega }\times r_{n},$$

where “<>” denotes averaging over the current and closest neighboring cells, $n_{\omega }$ is the unit vector aligned with the vorticity vector, and $r_{n}$ denotes the location vectors of the cell’s vertices. The scale ${\tilde{\Delta }}_{\omega }$ can be regarded as the larger scale of the projected grid on the plane perpendicular to the local vector, as shown in Table 1.

The Kelvin–Helmholtz instability un-locker $F_{KH}$ and vortex tilting measure in Equation (8) are defined as Equations (11) and (12), respectively.

(11) $$F_{KH}\left(<VTM>\right)=\max\left\{F_{KH}^{\min},\min\left\{F_{KH}^{\max},F_{KH}^{\min}+\frac{F_{KH}^{\max}-F_{KH}^{\min}}{a_{2}-a_{1}}\left(<VTM>-a_{1}\right)\right\}\right\},$$

(12) $$VTM=\frac{\sqrt{6}\left|\left(\hat{S}\cdot \omega \right)\times \omega \right|}{\omega^{2}\sqrt{3tr\left({\hat{S}}^{2}\right)-{\left[tr\left(\hat{S}\right)\right]}^{2}}}\max\left\{1,\left(\frac{v^{*}}{v_{t}}\right)\right\},\;v^{*}=0.2v,$$

where $F_{KH}^{\max}$ = 1.0, $F_{KH}^{\min}=0.1$, $a_{1}=0.15,$ and $a_{2}=0.3$. If the vorticity vector is parallel to the eigenvector of the strain tensor, then the VTM will be equal to 0, which means that the strain will only have a stretching or compression effect on the vorticity, corresponding to quasi-2D flow regions. When the vorticity vector is tilted toward other directions, VTM will not be 0, which means that the strain will make the vorticity develop in different directions, corresponding to developed 3D turbulence regions. This feature of VTM can realize the ability to reduce the grid length scale in quasi-2D flow regions but remain the normal grid length scale in the developed 3D turbulence regions.

It should be noted that VTM will also treat the RANS boundary layers as quasi-2D flow regions, which will decrease the local grid length scale and, thus, increase the burden of the shielding function. This will increase the risk of MSD. In some studies [19], the shielding function $f_{d}$ was used to inactivate the function $F_{KH}$ in the RANS region of the boundary layer, making the grid length scale fall back to ${\tilde{\Delta }}_{\omega }$ rather than 0.1${\tilde{\Delta }}_{\omega }$. In this study, the second shielding function $f_{P}$ is used to ensure that the shielding of the entire boundary layer is not affected by the local grid length scale, which overcomes this problem.

### 2.2. Modifications of the Shielding Function

In this section, the standard shielding function [23] and the second shielding function [3] are introduced, on the basis of which the modified shielding function $f_{VTM}$ is defined.

#### 2.2.1. Standard Shielding Function $f_{d}$

In the SST k–ω-based DDES model [23], the standard shielding function is defined as

(13) $$f_{d}\left(r_{d}\right)=1-\tanh\left[{\left(C_{1}r_{d}\right)}^{C_{2}}\right],\;C_{1}=3\;C_{2}=20,$$

(14) $$r_{d}=\frac{v_{t}+v}{k^{2}d_{w}^{2}\sqrt{0.5\cdot \left(S^{2}+\Omega^{2}\right)}}.$$

#### 2.2.2. Second Shielding Function $f_{P}$

The second shielding function $f_{P}$ proposed by Deck [3] for the ZDES mode2 (EP) is given by Equation (15).

(15) $$f_{P}=f_{d}\left(r_{d}\right)\left(1-\left(1-f_{P2}\right)f_{R}\left(G_{\Omega }\right)\right),$$

(16) $$f_{P2}=f_{d}\left(G_{\tilde{v}}\right),$$

(17) $$G_{\tilde{v}}=\frac{C_{3}\max\left(0,-\partial v_{t}/\partial n\right)}{\sqrt{u_{i,j}u_{i,j}}kd_{w}},\;C_{3}=25,$$

(18) $$f_{R}\left(G_{\Omega }\right)=\left\{\begin{array}{c} 1\;if\;G_{\Omega }\leq C_{4},\\ \frac{1}{1+\exp\left(\frac{-6\alpha }{1-\alpha^{2}}\right)}\;if\;C_{4}<G_{\Omega }\leq \frac{4}{3}C_{4},\;\;\alpha =\frac{\frac{7}{6}C_{4}-G_{\Omega }}{\frac{1}{6}C_{4}}\;C_{4}=0.03,\\ 0\;if\;G_{\Omega }\geq \frac{4}{3}C_{4}, \end{array}\right.$$

(19) $$G_{\Omega }=\frac{\partial \left(\left|\omega \right|\right)}{\partial n}\sqrt{\frac{v_{t}}{{\left(\sqrt{u_{i,j}u_{i,j}}\right)}^{3}}},$$

where $G_{\tilde{v}}$ is a dimensionless function reflecting the variation law of the eddy viscosity in the normal direction of the wall. $G_{\tilde{v}}$ mainly draws upon the characteristic that the wall-normal gradient of the eddy viscosity is negative in the outer area of the wall boundary layer and is positive or close to 0 in other areas. Then, the outer layer of the boundary layer is shielded by $f_{P2}$. It is assumed that the wall-normal gradient of |ω| will be positive between the separating location and the separating vortex core. The dimensionless function $G_{\Omega }$ constructed from this idea is used to detect the massively separating area and help $f_{R}\left(G_{\Omega }\right)$ inhibit the second shielding function $f_{P}$ to prevent excessive shielding, maintaining the LES behavior.

#### 2.2.3. Modified Shielding Function $f_{\mathrm{VTM}}$

Considering that the VTM can detect the 3D vortex structure, a new inhibition function $f_{RKH}$ constituted by VTM is constructed and superimposed on the second shielding function $f_{P2}$, contributing to the modified shielding function $f_{VTM}$ as per Equation (20).

(20) $$f_{VTM}=f_{d}\left(r_{d}\right)\left(1-\left(1-f_{P2}\right)f_{R}\left(G_{\Omega }\right)f_{RKH}\right),$$

(21) $$f_{RKH}=1-\max\left\{0,\min\left\{1,\frac{1}{a_{2}-a_{1}}\left(<VTM>-a_{1}\right)\right\}\right\},\;a_{1}=0.15,a_{2}=0.2,$$

(22) $$VTM=\frac{\sqrt{6}\left|\left(\hat{S}\cdot \omega \right)\times \omega \right|}{\omega^{2}\sqrt{3tr\left({\hat{S}}^{2}\right)-{\left[tr\left(\hat{S}\right)\right]}^{2}}},$$

where “<>” denotes averaging over the current and closest neighboring cells. $a_{1}$ and $a_{2}$ are threshold values that are adjustable empirical parameters, which are adjusted to 0.15 and 0.2, respectively, in the 2D hump case. $f_{RKH}$ is set as 1 in the RANS region and in the LES region without three-dimensional turbulence, in which case $f_{P2}$ and $f_{R}\left(G_{\Omega }\right)$ are activated as normal. When located in the LES region with strong 3D turbulence (area A in Figure 1), $f_{RKH}$ is set to 0, which makes $f_{P2}$ invalid and reduces the RANS areas shielded by $f_{VTM}$. It should be noted that in the separated and reattached regions, the shielding function $f_{VTM}$ still remains at the level of the standard shielding function $f_{d}\left(r_{d}\right)$ in order to prevent the RANS/LES interface from getting too close to the wall surface, in which case a large number of grids will be required to support the LES.

## 3. Results

### 3.1. Numerical Methodology

All tests were performed using the open-source CFD environment OpenFOAM, which is a cell-centered, unstructured, finite-volume-based code of second-order accuracy in temporal and spatial discretization. Some studies on variants of the DDES model have been carried out for separated flows, free shear flows, etc. using OpenFOAM [11,14], proving the reliability of the DDES code in OpenFOAM. All the calculations in this paper were performed with the incompressible solver [25]. The second-order central difference is used in the LES area because it is non-dissipative and conservative, whereas the upwind scheme is not used in the LES area because of too much numerical dissipation [26]. The hybrid convection scheme of Travin et al. [27] was used to adopt the robust second-order upwind differencing in the RANS area and low-dissipation second-order central differencing in the LES area. In order to ensure computational convergence, all cases were first run with the steady SST-RANS and were then switched to the unsteady SST-DDES with turbulent transport equations marching but N–S equations frozen. After the convergence of turbulent transport equations, the unsteady calculation of SST-DDES returned to normal.

### 3.2. Flat-Plate Boundary-Layer Case

The flat-plate boundary-layer case was used to verify whether the shielding function $f_{VTM}$ could guarantee RANS behavior in the entire boundary layer regardless of the infinitely fine meshes, which might mitigate the MSD problem.

**Figure 2:**
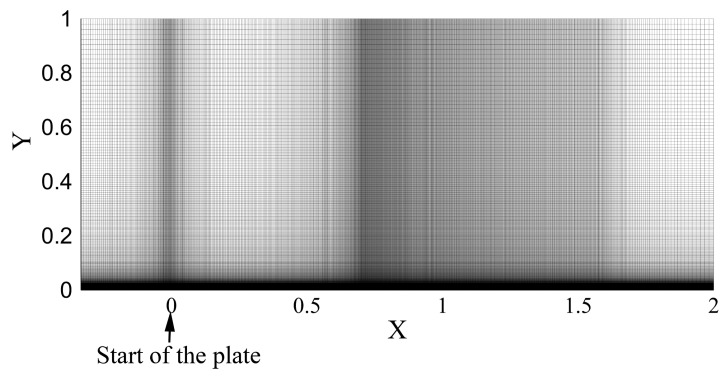
Computational domain and grids.

**Figure 3:**
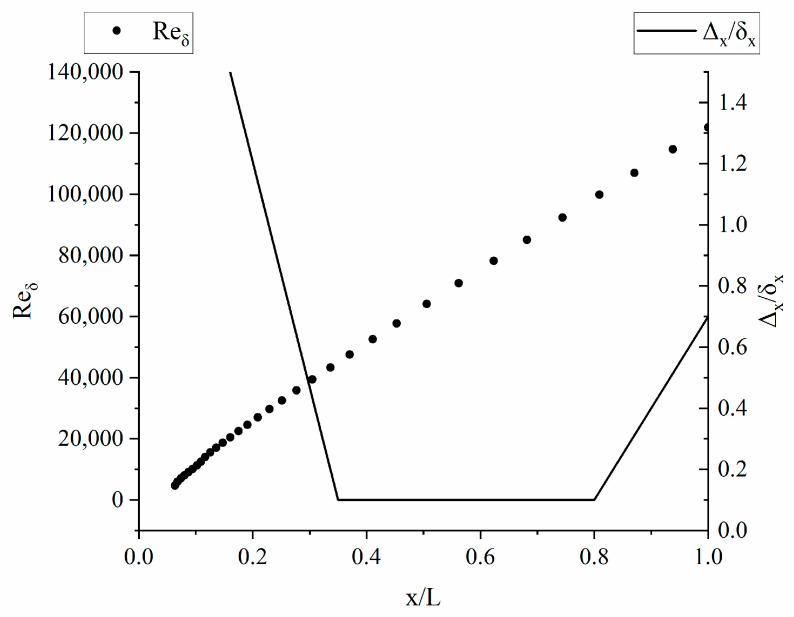
Grid length $\Delta_{x}$/$\delta_{x}$ and boundary thickness Reynolds number in the X-direction.

This case was set as a zero-pressure gradient turbulent boundary layer over a flat plate of L = 2 m. The free stream velocity was $U_{\infty }$ = 69.4 m/s, leading to a Reynolds number per meter Re = $5\times 10^{6}$ $m^{-1}$. The computational domain and grids in the XY-plane are shown in Figure 2. The plate started at x = 0, before which a symmetry boundary was set. The grids in the X direction were set according to the flat-plate case by Deck [3], i.e., keeping $\Delta_{x}$/$\delta_{x}$ ≈ 0.1 in the area 0.35 ≤ x/L ≤ 0.8, which was considered the “challenging area”, while setting coarser X-direction grids in other areas (Figure 3). In the Y-direction (wall-normal direction), $y^{+},$ based on the mesh center of the first layer, was set to approximately 1.0. The grids in the Z-direction were set to 0.5$\Delta_{x}$ at x/L = 0.35, where $\Delta_{x}$ was the smallest. The grid numbers in all directions were $N_{x}\;$= 1053, $N_{y}$ = 385, and $N_{z}$ = 10, leading to four million cells in total.

**Figure 4:**
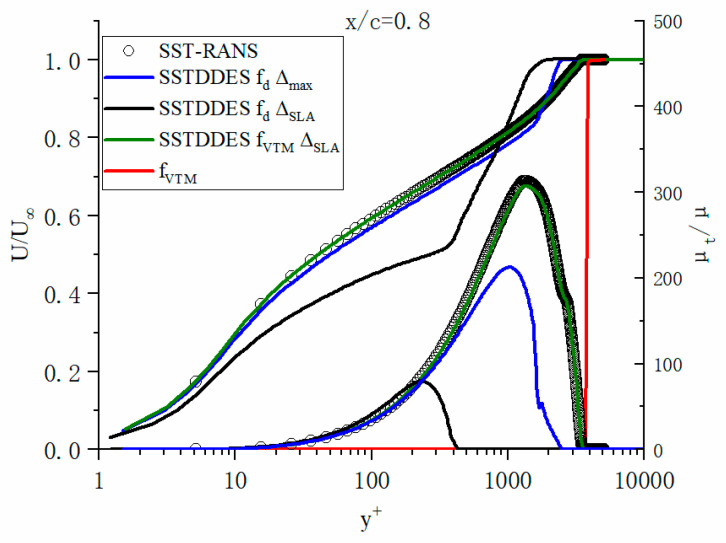
Velocity, eddy viscosity, and $f_{VTM}$ profiles at x/L = 0.8.

The profiles of the velocity, eddy viscosity, and $f_{VTM}$ in Figure 4 show that $f_{VTM}$ inherited the advantage of $f_{P}$ and succeeded in shielding the whole boundary layer, keeping the results highly similar to those for RANS. Serious MSD in the challenging area was shown in the DDES with the standard shielding functions $f_{d}$ and $\Delta_{max}$. When the SLA grid scale $\Delta_{SLA}$ was applied, the MSD problem for the $f_{d}$ DDES was seriously aggravated in the challenging area owing to a decrease in the local grid length scale in the boundary layer. This proves that $\Delta_{SLA}$ weakened the ability of the standard shielding function in shielding the whole boundary layer. The $f_{d}$ $\Delta_{SLA}$ DDES behaved like the WMLES at x/L > 0.65, with modeled turbulence switching into resolved turbulence, which still could not alleviate the MSD. However, the second shielding function $f_{P}$ alleviated the MSD to the greatest extent even when $\Delta_{SLA}$ was applied, verifying the superiority of the shielding capacity.

### 3.3. Flow over the Hump

The flow over the hump in this paper refers to the NASA wall-mounted hump separated flow validation experiment [28]. It includes the representative separation phenomenon induced by adverse pressure gradients, often occurring in suction surfaces of turbomachinery fans, compressor blades, etc. Accurate prediction for the reattachment of a separated flow is difficult for both RANS and DDES. A test was conducted to contrastively examine the overall performance of the MSRT DDES and other variants of DDES.

**Figure 5:**
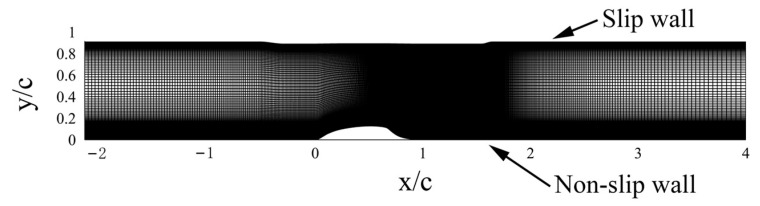
Computational domain and grid in XY-plane.

The computational domain is shown in Figure 5. The hump cord length, c, was equal to 0.42 m. The nominal test section height (between the splitter plate and the upper wall) was 0.382 m, equal to 0.91c. The width in the spanwise direction was 0.4c, referring to the previous case setting in the Go4Hybrid project [11]. The inflow velocity of the mainstream was U = 34.6 m/s, leading to a Reynolds number of $9.36\times 10^{5}$ based on the hump chord c.

The hump and splitter plate are considered to be no-slip wall conditions. The upper wall includes a contour to its shape to approximately account for the blockage caused by the end plates in the experiment, which uses slip wall conditions. The inflow boundary is set at x/c = −2.14, with a fully turbulent boundary layer of 0.08c thickness. The velocity and turbulent quantity profiles at the inflow boundary were obtained from a steady k–ω SST RANS of the zero-pressure gradient boundary layer over a flat plate, which was set to match the profiles from the experiment at x/c = −2.14. The boundary conditions in the spanwise direction were periodic.

**Figure 6:**
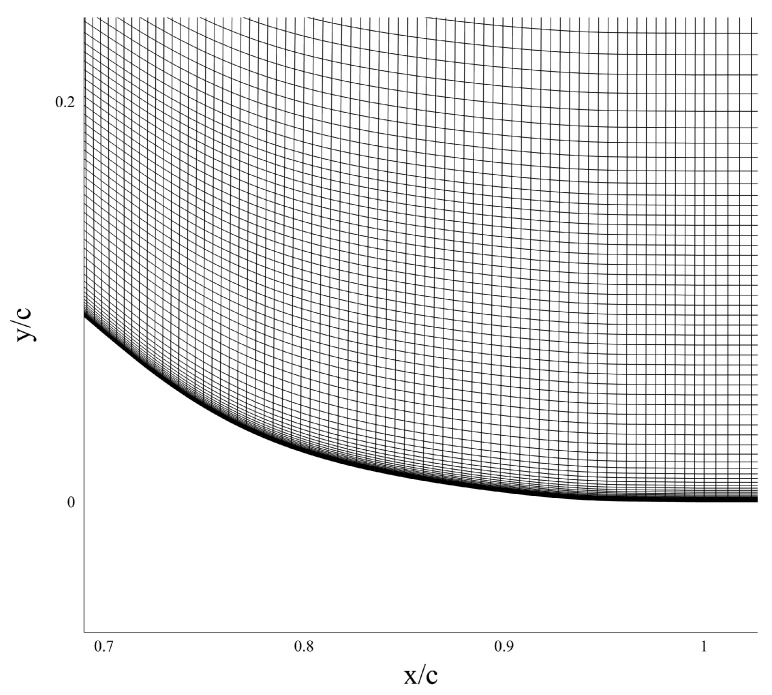
“Focus region” grid in the XY-plane.

The structured grids from the Go4Hybrid project [11] in the XY-plane are shown in Figure 5. The first-layer grid $y^{+}$ was set to be less than 1. The separated and reattached region (0.67 < x/c < 1.5) was considered the “focus region” in Figure 6, keeping $\Delta_{x}$ and $\Delta_{y}$ approximately equal to 0.005c. The spanwise grid width was constantly equal to 0.005 c, contributing to a fine grid resolution for the LES branch in the “focus region”. The total grid number was 511 × 127 × 80 ≈ 5.2 million. The timestep was set to dt =$\;2\times 10^{-5}$ s.

**Figure 7:**
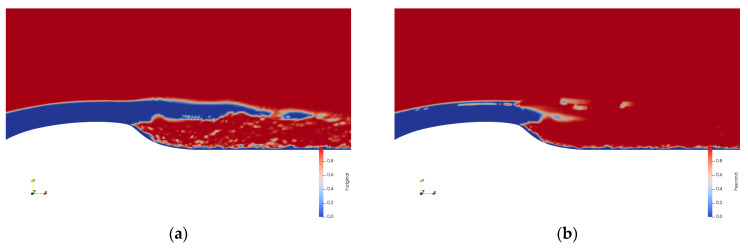
Contours of shielding function: (**a**) $f_{P}$; (**b**) $f_{VTM}$.

By observing the overall performance of the two shielding functions in Figure 7, it is clear that the second shielding function $f_{P}$ suffered from randomly returning to RANS regions (where $f_{d}$ = 0) in the LES region after the separated point. In contrast, the modified shielding function $f_{VTM}$ in this paper was more consistent with physical situations and maintained LES behavior in the whole region with resolved turbulence. The unreasonable behavior of the second shielding function $f_{P}$ in the separated and reattached area can be explained as follows: over the plate behind the hump, there were resolved turbulence fluctuations caused by flow separation upon the reattached boundary layer, which made the distribution of eddy viscosity in this area relatively random, further leading to a random distribution of the normal-wall gradient of eddy viscosity $\partial v_{t}/\partial n$. Moreover, this area was still close to the wall. Considering the above, the second shielding function sensor $G_{\tilde{v}}$, constituted by $\partial v_{t}/\partial n$ and the wall distance $d_{w}$ in Equation (17), led to the irregular activation of RANS behavior in LES regions near the boundary layer.

**Figure 8:**
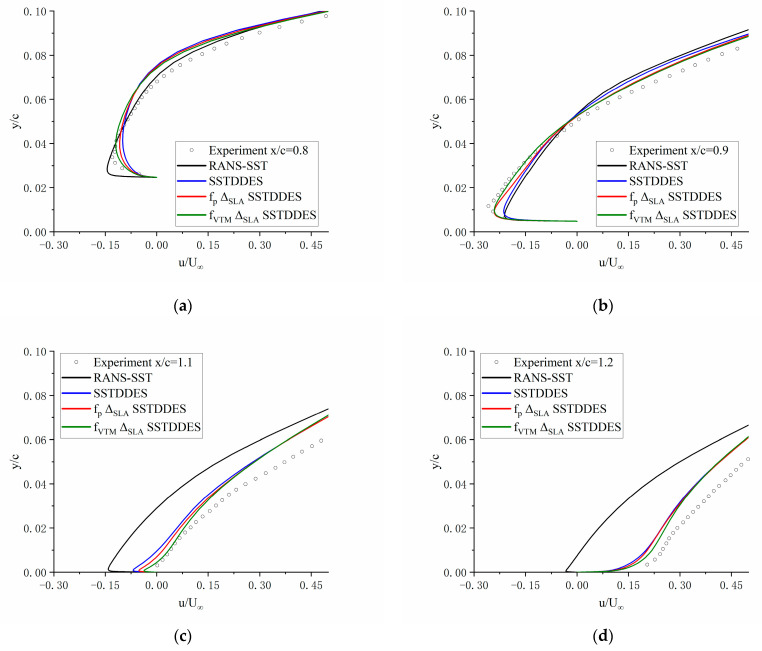
Velocity profiles at different locations: (**a**) x/c = 0.8; (**b**) x/c = 0.9; (**c**) x/c = 1.1; (**d**) x/c = 1.2.

Figure 8 shows that, at some locations where x/c < 1, the average RANS velocity profiles were closer to the experimental results than the DDES variants. However, when it comes to the separated and reattached area (x/c > 1), the average velocity profiles of each DDES variant were closer to the experimental values than RANS. In addition, the $f_{VTM}\;\Delta_{SLA}$ DDES was advanced in the development of the velocity from the negative gradient to the positive gradient, which had more consistency with the experimental result than the $f_{P}\;\Delta_{SLA}$ DDES. This indicates that $f_{VTM}$ displayed the advantages of LES in the separated–reattached region to a greater extent and reduced the negative impact existing in the RANS model under the precondition of grid support.

In summary, $f_{VTM}$ adopted the second shielding function before the separated point to ensure RANS behavior in the entire boundary layer. After separation, $f_{VTM}$ inactivated the second shielding function and returned to the standard shielding function. Compared with $f_{d}$ and $f_{P}$, the shielding strategy of $f_{VTM}$ proposed in this paper had a better result for the hump cases.

## 4. Corner Separation in the Linear Compressor Cascade

After two basic cases, the $f_{VTM}\;\Delta_{SLA}$ DDES had the best performance among the tested strategies, and it was referred to as the modified shielding and rapid transition DDES (MSRT DDES). Considering the fact that all the tested fluid phenomena involved two-dimensional separations, the MSRT DDES is applied to complex and three-dimensional separation in this section, which comprises the corner separation in the compressor cascade.

Corner separation occurs near the hub and suction surface of the compressor blade row, which is large-scaled and highly three-dimensional. Corner separation is induced by the strong adverse pressure gradient and secondary flow [20]. It will lead to total pressure loss, which limits the static pressure rise, reduces the compressor efficiency, and contributes to the passage blockage [29]. Therefore, it is of great significance to predict the corner separation correctly with a fairly low cost.

The linear compressor cascade from Ecole Centrale de Lyon is the benchmark case according to which numerical methods can be tested for corner separation [30,31,32,33,34]. Compared to the experiment in [31], LES agreed well with this experiment, while having a cost of 200 million grid points. Some RANS studies [35], including SA, k–ε, k–ω, and k–ω SST, overpredicted the total pressure loss and separation area. Xia applied a k–ω–SST-based DDES with $C_{2}$ = 8 instead of 20, and an $\Delta_{SLA}$ DDES with $C_{2}$ = 20 to the corner separation case, which both seemed to improve the total pressure loss compared to RANS and standard DDES. However, both solutions could suffer from severe MSD problems, especially when the grids near the leading edge are refined to capture the unsteadiness of a horseshoe vortex (HSV), which is considered an important element to indicate corner separation. The MSRT DDES has the potential to improve the behavior of DDES and overcome the MSD.

**Figure 9:**
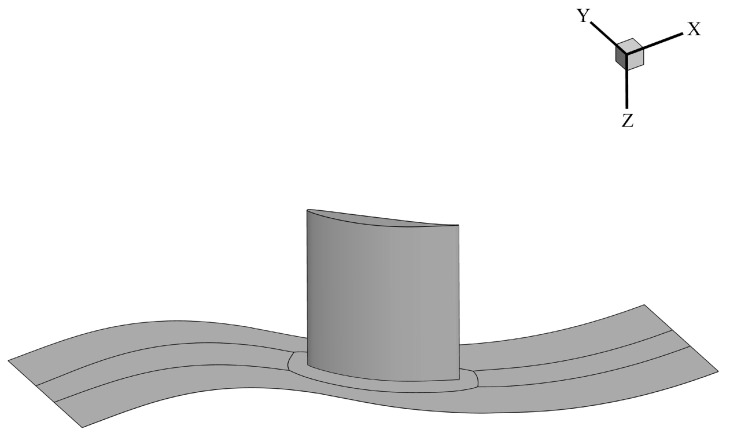
Computational domain of the cascade.

The key parameters of the linear cascade are shown in Table 2, which can be found in Ma et al. [31] in detail. The computation domain is shown in Figure 9, including a single passage and half-blade-span domain of the cascade. The inlet freestream velocity was $U_{\infty }=40\;m/s$. The inflow boundary was set at x/$c_{a}$ = −2.2 with the leading edge set as x/$c_{a}$ = 0, where the axial chord length $c_{a}$ was 0.11 m. A fully turbulent boundary RANS profile was set for the hub in the inlet, which was obtained using a flat-plate turbulent boundary layer RANS case to match the experimental data. The outflow boundary was set 2$c_{a}$ downstream of the trailing edge as suggested in Yin [36].

**Figure 10:**
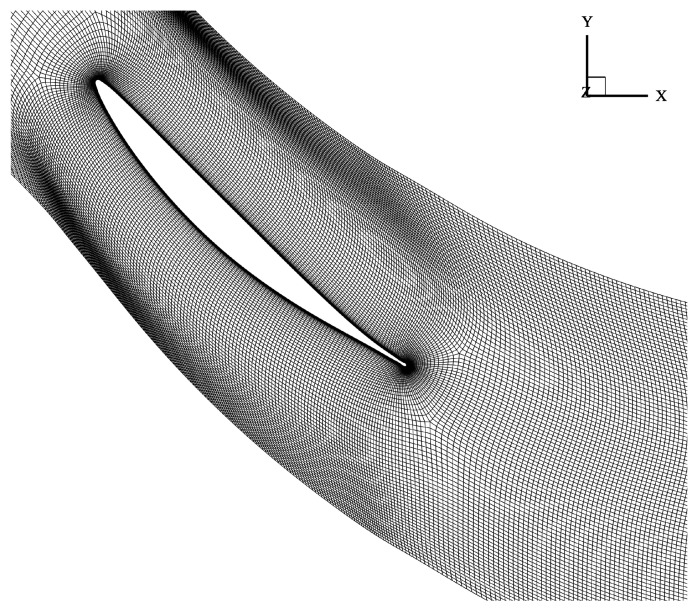
HOH mesh for the passage plane.

The HOH meshes were generated by AUTOGrid5 with an O-block around the blade and H-blocks for the passage, as shown in Figure 10. The spacing of the first-layer grid near the viscous walls was set to $2.5\times 10^{-6}$ m to guarantee $y^{+}<1$. The grids near the separation area were set to be orthotropic and isotropic for LES. Two grids were studied in this paper. Grid 1 contained a 336 × 50 O-grid and a 316 × 100 H-grid, with 100 layers in the spanwise direction, leading to 4.8 million grids; this arrangement was used in Yin [36]. The finer Grid 2 contained a 626 × 50 O-grid and a 508 × 204 H-grid, with 160 layers in the spanwise direction, leading to able 20 million grids; this arrangement had a higher grid resolution near the separation area. Since there is no concept of “mesh independence” in the LES region (because the behaviors of the sub-grid model are based on mesh scale changes), it is necessary to ensure that the mesh scale in the LES region is within the inertial range of the turbulent energy spectrum. A more relaxed mesh requirement is to set the scale in a range where the turbulent energy generation and dissipation have relatively small energy cascades. In general, the mesh scale is taken as l06~60η, where $l_{0}$ is the integral length scale and η is the dissipation scale. The relationship between the integral length scale and the dissipation scale is l0η~Rel03/4, where Rel0=ρu′l0μ, $u^{\prime }$ represents the turbulent fluctuating velocity. Both grids satisfy the scale requirement. Yin [36] compared three different grid resolutions for the same liner cascade case in detail, with 2.82 million, 4.84 million, and 9.60 million grids. The results showed that 9.60 million grids did not show significant improvement compared to 4.84 million grids [20,35]. In summary, Grid 1 and Grid 2 in our study satisfied the numerical requirement. 

The timestep in the unsteady computation was set to $d_{t}$ = $5\times 10^{-6}$ s. The unsteady result in 0.2 s of physical time was averaged and analyzed. The computation with Grid 2 took 274 h with 120 CPUs used in parallel.

**Figure 11:**
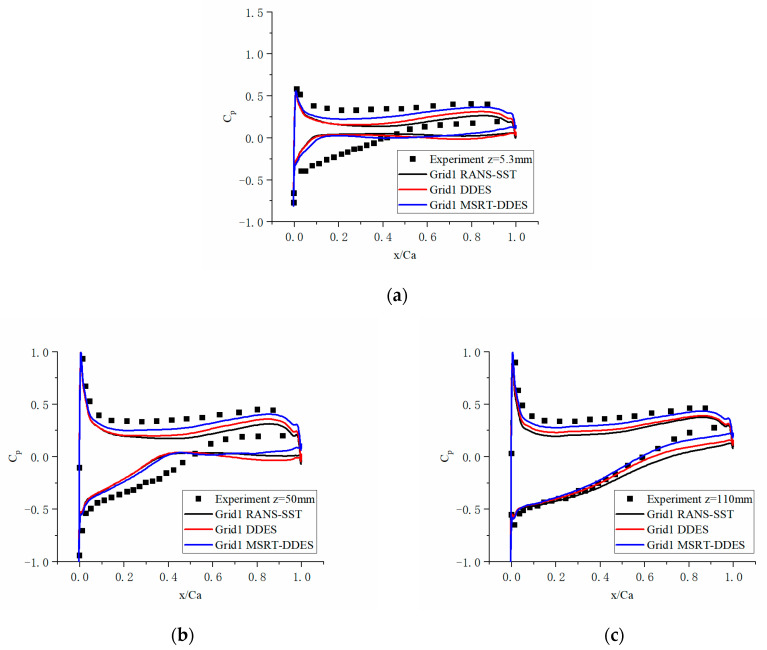
Static pressure coefficient $C_{p}$ at different spanwise positions for grid 1: (**a**) z/h = 1.4%; (**b**) z/h = 13.5%; (**c**) z/h = 29.7%.

Figure 11 shows the static pressure coefficient $C_{p}$ at z/h = 1.4%, 13.5%, and 29.7% for Grid 1. $C_{p}$ is defined as

(23) $$C_{p}=\frac{p-p_{\infty }}{\frac{1}{2}\rho U_{\infty }^{2}}.$$

**Figure 12:**
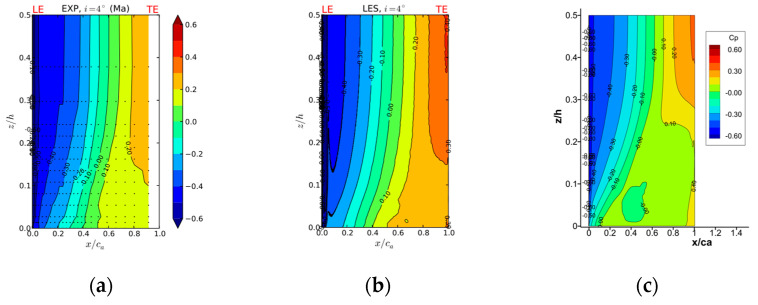
Static pressure coefficient $C_{p}$ at the suction surface: (**a**) Exp; (**b**) LES; (**c**) MSRT DDES.

Around the blade surface at all three spanwise positions, $C_{p}$ for the MSRT DDES was closer to the experimental data than k–ω SST RANS and standard DDES, especially for the whole pressure surface and the downstream part of the suction surface. When close to the trailing edge, the resolved turbulence became fully developed owing to the acceleration of $\Delta_{SLA}$, which led to a better result than the standard DDES. However, for the suction surface near the leading edge, $C_{p}$ at z/h = 1.4% and 13.5% almost had the same deviation from the experiment for three different models. The constant pressure area ($C_{p}\approx 0$) resulting from corner separation was more ahead than the experimental value, which can be seen more clearly from the $C_{p}$ distribution on the suction surface in Figure 12.

The total pressure-loss coefficient distribution is defined as

(24) $$C_{pt}=\frac{p_{t,\infty }-p_{t}}{p_{t,\infty }-p_{s,\infty }}.$$

**Figure 13:**
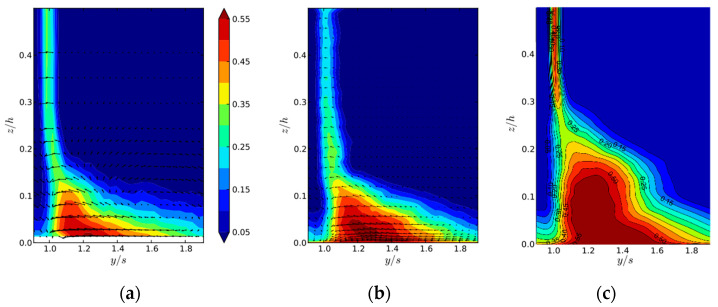
Total pressure-loss coefficient distribution $C_{pt}$ at $x/c_{a}=1.363$ for Grid 1: (**a**) Exp; (**b**) LES; (**c**) MSRT DDES.

**Figure 14:**
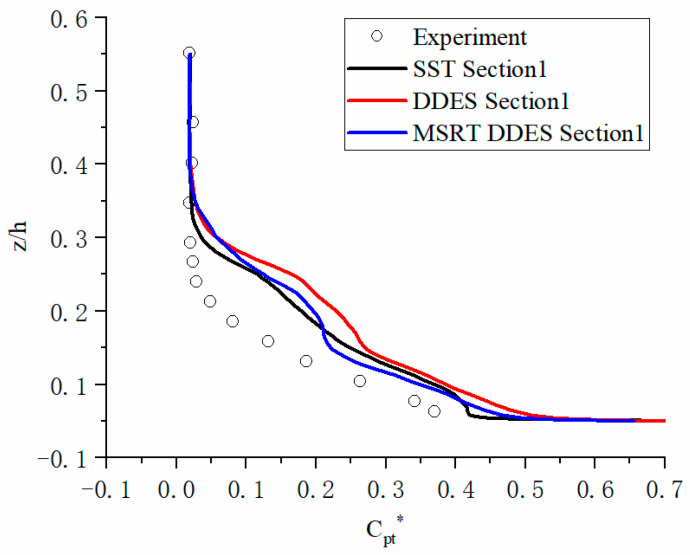
Pitchwise integrated total pressure loss $C_{pt}^{*}$ at $x/c_{a}=1.363$ for Grid 1.

**Figure 15:**
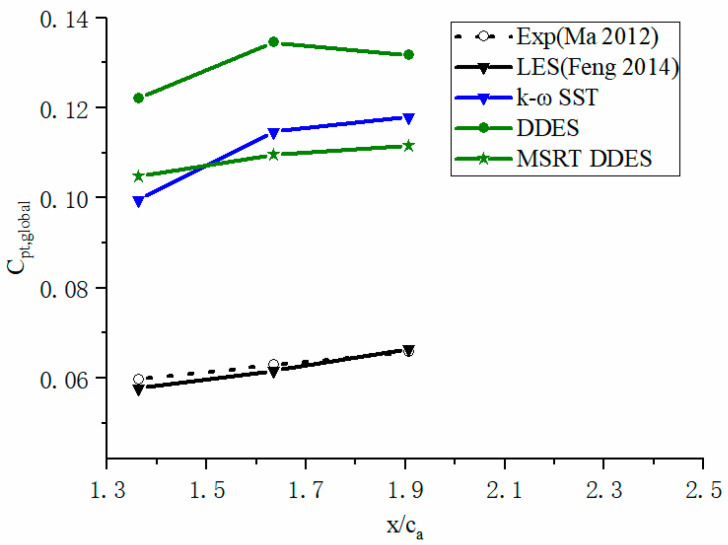
Overall integral total pressure loss $C_{pt,\mathrm{global}}$ along the axial direction $x/c_{a}$ for Grid 1 [30,33].

Figure 13 shows $C_{pt}$ at $x/c_{a}=1.363$. The larger area of high total pressure loss for the MSRT DDES resulted from the earlier and larger corner separation compared to upstream. Figure 14 and Figure 15 show the pitchwise integrated total pressure loss $C_{pt}^{*}$ along the blade span at $x/c_{a}=1.363$ and the overall integral total pressure loss $C_{pt,\mathrm{global}}$ along the axial direction, which are defined as

(25) $$C_{pt}^{*}\left(z\right)=\frac{\int_{0}^{s}C_{pt}\left(y,z\right)u\left(y,z\right)dy}{\int_{0}^{s}u\left(y,z\right)dy}.$$

The total pressure loss for the MSRT DDES was closer to the experimental value than the standard DDES, while it was almost at the same level as the RANS.

According to the above comparison, the MSRT DDES performed better than the standard DDES. The result for the MSRT DDES was improved by predicting the separating location and separating scale more precisely. However, the best loss result obtained via a hybrid simulation was almost equal to that obtained via a pure RANS simulation, as shown in Figure 15, because the point of separation and the extension of the separation zone were determined by the RANS part of the modeling. 

**Figure 16:**
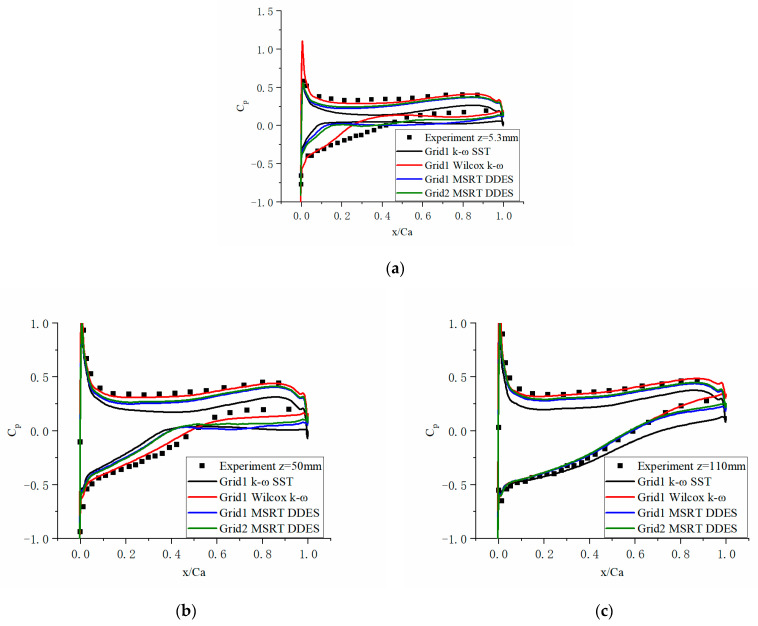
Static pressure coefficient $C_{p}$ at different spanwise positions: (**a**) z/h = 1.4%; (**b**) z/h = 13.5%; (**c**) z/h = 29.7%.

**Figure 17:**
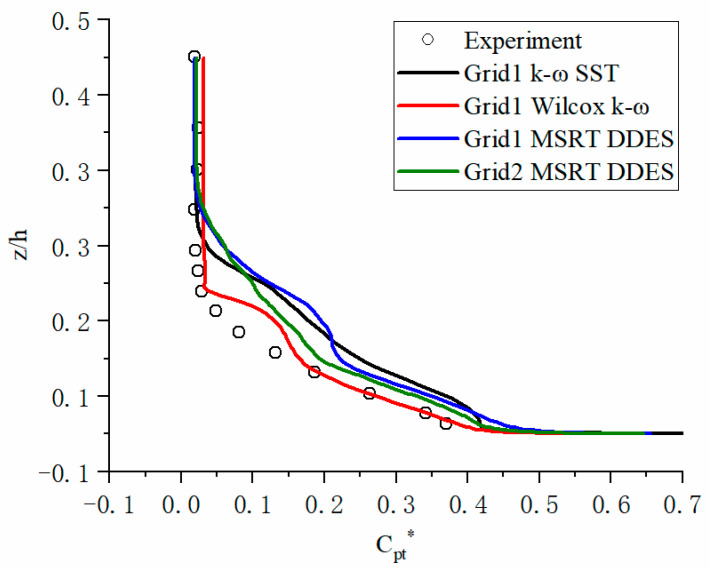
Pitchwise integrated total pressure loss $C_{pt}^{*}$ at $x/c_{a}=1.363$.

**Figure 18:**
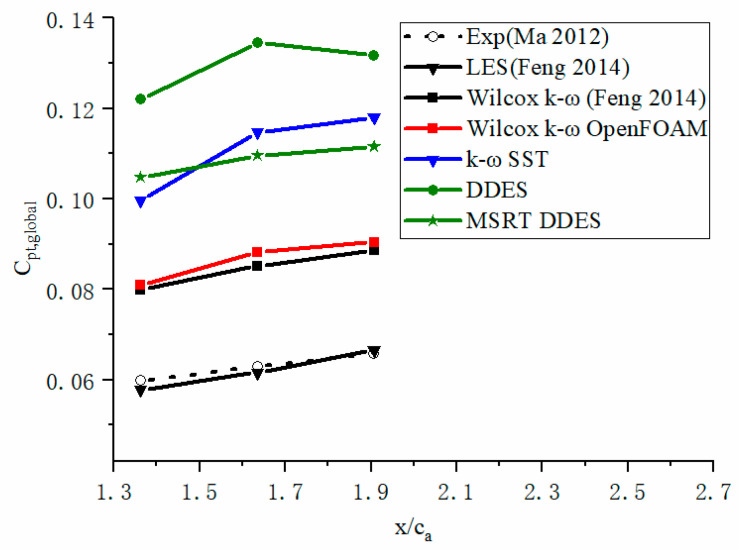
Overall integral total pressure loss $C_{pt,\mathrm{global}}$ along the axial direction $x/c_{a}$ [30,33].

The LES grid resolution and the RANS behavior of DDES might have an influence on the separation. Grid 2, with a finer LES grid, was used to study the impact on the corner separation. In addition, the Wilcox k–ω RANS [37] was used to study the impact of RANS models on the separation location. As shown in Figure 16, the difference in $C_{p}$ for the MSRT DDES between Grid 1 and Grid 2 was limited, while $C_{p}$ for the Wilcox k–ω RANS is much closer to the experimental value. In Figure 17 and Figure 18, the integrated total pressure loss for the Grid 2 MSRT DDES had a moderate reduction compared to Grid 1 MSRT DDES, while that for the Wilcox k–ω RANS was much closer to the level of the experiment (the Wilcox k–ω RANS result from Feng [32,33] is plotted for comparison in the Figure 18, proving the reliability of the RANS result). This is direct proof of the argument that the RANS part of the modeling determined the quality of the prediction of the loss for the corner separation to the largest extent. The loss result of a DES cannot be better than the loss result of the basic RANS for corner separation.

Corner separation can also be better characterized by entropy production. There are various ways to calculate entropy production (ΔS); in physical and thermodynamics, it can generally be related to the internal energy (U) of a system:

(26) dU=T dS−p dV.

For incompressible substances, where the change in volume (V) is considered to be zero, the entropy is only a function of temperature (T):

(27) TdS=dU=cdT.

Then, the entropy production can be defined as

(28) $$\Delta S=\int c\frac{dT}{T}.$$

In order to get rid of this dependence on freestream Mach numbers, the entropy increment ratio ($S_{ratio}$) is introduced as follows [38]:

(29) Sratio=SS∞−1Ma∞.

**Figure 19:**
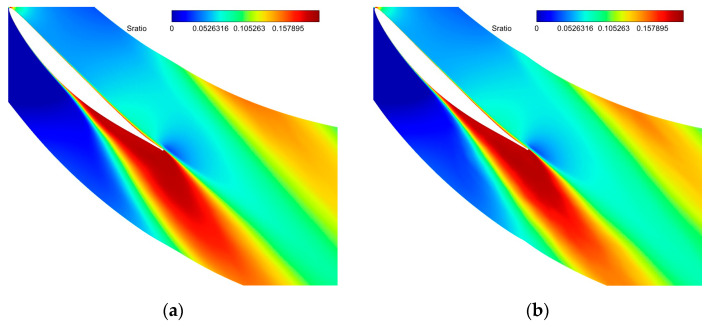
Entropy increment ratio $S_{ratio}$ at z/h=13.5%: (**a**) grid 1 MSRT DDES; (**b**) grid 2 MSRT DDES.

The finer LES grid reduced the numerical viscosity from spatial discretization, contributing to the acceleration of the transition. The higher level of resolved turbulence in Grid 2 reduced the width of the separation region in the vertical direction of the main flow compared to Grid 1, as shown in Figure 19. However, the starting point of the separation was almost unchanged when the grid was refined. Furthermore, the choice of RANS model led to a significant difference in both the separation point and the scale of the separation region. This proves that the choice of the RANS turbulence model was also the determining factor for the separation point. The Wilcox k–ω RANS considered the whole boundary layer as fully developed turbulence from the leading edge, which reinforces the ability of anti-separation. This delayed the corner separation and reduced the total pressure loss. Considering that two tripping wires were set on the leading edge to strengthen the turbulence in the experiment, the Wilcox k–ω-based DDES was expected obtain a more reasonable result than the k–ω SST-based DDES.

In summary, the performance of the MSRT DDES was more successful than the standard DDES and the closest to the corresponding RANS. The remaining gap between the MSRT DDES and the experimental results can be attributed to the poor performance of the k–ω SST branch. Therefore, the choice of the RANS branch in the DDES plays an important role for the prediction of corner separation.

## 5. Conclusions

In this study, we proposed a version of DDES with modified shielding and rapid transition (MSRT DDES). In the modified shielding function $f_{VTM}$, an inhibition function $f_{RKH}$ based on the VTM was constructed to inactivate the second shielding function adaptively when resolved turbulence was induced by the separated flow. This was the first attempt to apply the VTM to the shielding function, and it worked as expected in these cases. The VTM was reutilized in the $\Delta_{SLA}$ to accelerate the transition from RANS to LES.

- The $\Delta_{SLA}$ provided practical remedies to the RANS-LES transition problem and maintained normal LES behavior in the developed 3D turbulence. Reutilization of the VTM made the increase of computational consumption acceptable.
- The modified shielding function $f_{VTM}$ was successful in solving the MSD problem, which could be exacerbated by mesh refinement and the utilization of $\Delta_{SLA}$.
- The original second shielding function $f_{P}$ was found to make DDES abnormally switch to RANS behavior when resolved turbulence was present in separated and reattached flow near the wall, which was ameliorated by the introduction of the new inhibition function $f_{VTM}$. The utilization of $f_{VTM}$ led to a moderate improvement with respect to separated–reattached flows.
- The behavior of the MSRT DDES was more reliable than the standard DDES for a three-dimensional separation flow. By conducting a detailed analysis of physical quantities such as entropy increment ratio and total pressure-loss coefficients, the loss result of the MSRT DDES was almost equal to that of the corresponding RANS, which is proof of success when considering that the point of separation and the extension of the separation zone were mostly determined by the RANS part of the modeling in this case. The performance of the MSRT DDES could be further improved by the proper selection of the RANS base, e.g., the Wilcox k–ω-based MSRT DDES. This will be developed and evaluated in future work.

In conclusion, the MSRT DDES makes use of the second shielding function $f_{P}$ and $\Delta_{SLA}$ to ameliorate the main issues present in DES-type models, while overcoming some of the deficiencies with respect to $f_{P}$ and $\Delta_{SLA}$. This DDES variant has the potential to obtain promising results in more complex situations, such as the tip leakage flow in compressor blades. The MSRT DDES with different RANS bases and for more complex cases will be evaluated in future work.

## Figures and Tables

**Table 1:** Order of grid length scale in different regions.

Grid Length Scale	Quasi-2D Flow Regions	Developed 3D Turbulence
$\Delta_{\max}$ [23]	$O\left(\Delta z\right)$	$O\left(\max\left(\Delta x,\Delta y,\Delta z\right)\right)$
$\Delta_{\mathrm{cube}-\mathrm{root}}$ [24]	$O\left(\sqrt[{3}]{\Delta x\Delta y\Delta z}\right)$	$O\left(\sqrt[{3}]{\Delta x\Delta y\Delta z}\right)$
${\tilde{\Delta }}_{\omega }$ [18]	$O\left(\max\left(\Delta x,\Delta y\right)\right)$	$O\left(\max\left(\Delta x,\Delta y,\Delta z\right)\right)$
$\Delta_{SLA}$ [18]	$0.1\cdot O\left(\max\left(\Delta x,\Delta y\right)\right)$	$O\left(\max\left(\Delta x,\Delta y,\Delta z\right)\right)$

**Table 2:** Key parameters of the linear cascade.

Name	Magnitude
Chord (c)	0.150 m
Pitch/spacing (s)	0.134 m
Blade span (h)	0.370 m
Stagger angle (γ)	42.70°
Camber angle (φ)	23.22°
Incidence angle (i)	4°

## Data Availability

Not applicable.
